# Retrieval of Recent Autobiographical Memories is Associated with Slow-Wave Sleep in Early AD

**DOI:** 10.3389/fnbeh.2013.00114

**Published:** 2013-09-18

**Authors:** Géraldine Rauchs, Pascale Piolino, Françoise Bertran, Vincent de La Sayette, Fausto Viader, Francis Eustache, Béatrice Desgranges

**Affiliations:** ^1^U1077, INSERM, Caen, France; ^2^UMR-S1077, Université de Caen Basse-Normandie, Caen, France; ^3^UMR-S1077, Ecole Pratique des Hautes Etudes, Caen, France; ^4^U1077, Centre Hospitalier Universitaire, Caen, France; ^5^Laboratoire Mémoire et Cognition, Institut de Psychologie, Université Paris Descartes, Paris, France; ^6^Centre de Psychiatrie et Neurosciences, INSERM UMR S894, Paris, France; ^7^Service des Explorations Fonctionnelles Neurologiques, Centre Hospitalier Universitaire, Caen, France; ^8^Service de Neurologie, Centre Hospitalier Universitaire, Caen, France

**Keywords:** Alzheimer’s disease, sleep, autobiographical memory, memory consolidation, PET

## Abstract

Autobiographical memory is commonly impaired in Alzheimer’s disease (AD). However, little is known about the very recent past which is though highly important in daily life adaptation. In addition, the impact of sleep disturbances, also frequently reported in AD, on the consolidation, and retrieval of autobiographical memories remains to be assessed. Using an adaptation of the TEMPau task, we investigated the neural substrates of autobiographical memory for recent events and the potential relationship with sleep in 14 patients with mild AD. On day 1, autobiographical memory was explored across three periods: remote (18–30 years), the last 2 years and the last month. After testing, sleep was recorded using polysomnography. The next day, AD patients benefited a resting-state ^18^FDG-PET scan and a second exploration of autobiographical memory, focusing on the very recent past (today and yesterday). Total recall and episodic recall scores were obtained. In addition, for all events recalled, Remember responses justified by specific factual, spatial, and temporal details were measured using the Remember/Know paradigm. Retrieval of autobiographical memories was impaired in AD, but recall of young adulthood and very recent events was relatively better compared to the two intermediate periods. Recall of recent events (experienced the day and the day preceding the assessment) was correlated with brain glucose consumption in the precuneus and retrosplenial cortex, the calcarine region, the angular gyrus, and lateral temporal areas. AD patients also provided more Justified Remember responses for events experienced the previous-day than for those experienced the day of the assessment. Moreover, Justified Remember responses obtained for events experienced before sleep were positively correlated with the amount of slow-wave sleep. These data provide the first evidence of an association between the ability to retrieve recent autobiographical memories and sleep in mild AD patients.

## Introduction

Autobiographical memory is a multifaceted concept which concerns information and experiences of one’s personal life and gives a sense of self-continuity (Piolino et al., 2009). Based on the observation of the amnesic patient KC, Tulving et al. (1988) proposed to distinguish within autobiographical memory an episodic component (altered in KC), containing personal specific events, situated in time and space (‘*the day I had this car accident*’), and a semantic component, termed personal semantic memory (preserved in KC), storing general knowledge about our past such as the names of colleagues, generic events (‘*summer holidays at Sainte Marine*’), and self-concepts. Autobiographical memory is subserved by a core neural network, mainly left-sided, including the medial and ventrolateral prefrontal cortex, medial, and lateral temporal areas, the retrosplenial/posterior cingulate cortex, the temporoparietal junction, and the cerebellum (Maguire, 2001; Conway et al., 2002; Svoboda et al., 2006; Cabeza and St Jacques, 2007; Martinelli et al., 2013a).

In the present study, we were interested in the assessment of recent episodic autobiographical memories and in the brain areas supporting their retrieval. We also investigated the impact of sleep on memory recall.

Numerous studies documented a general impairment of autobiographical memory in Alzheimer’s disease (AD) (Kopelman et al., 1989; Greene and Hodges, 1996; Addis and Tippett, 2004; Ivanoiu et al., 2004; Irish et al., 2011). Most of them showed a temporally graded retrograde amnesia, with memories from the young adulthood period being better preserved than recent ones (a phenomenon well-known as the “reminiscence bump,” Rubin et al., 1986). By distinguishing both components of autobiographical memory, a dissociation emerged in AD. Thus, Piolino et al. (2003), using a semi-structured questionnaire (TEMPau) assessing the ability to recollect detailed specific events situated in time and space from different periods covering the entire lifespan (from childhood/teenage to the last 12 months), they showed that retrieval of strictly episodic autobiographical memories (i.e., unique, specific in time and space, and detailed) is impaired in AD patients whatever the time period. Patients also exhibited a deficit of autonoetic consciousness, defined as the feeling of re-experiencing or reliving the past and mentally traveling back in subjective time. Contrasting with this impairment of episodic autobiographical memories, Martinelli et al. (2013b) documented a preservation of personal semantic memory assessed by asking AD patients to recall generic events memory. However, other studies reported a temporally graded deficit of personal semantic memory, with a relative preservation of the most recent information (Kopelman et al., 1989; Addis and Tippett, 2004). Greene et al. (1995) reported a similar deficit whatever the time period explored while Ivanoiu et al. (2004) observed a deficit with only a modest temporal gradient.

Several authors tried to disclose the brain areas whose atrophy or dysfunction may explain the impairment of autobiographical memory in AD. Thus, Gilboa et al. (2005) reported that episodic autobiographical memory was associated with the volume of the medial temporal lobes and the anterior lateral temporal neocortex. Interestingly, this pattern of correlation was invariant whatever the remoteness of memories suggesting that the integrity of medial temporal lobes is crucial for the retrieval of episodic autobiographical memories regardless of their age. In contrast, deficits of personal semantics were related to the atrophy of anterior and posterior lateral temporal areas bilaterally, more pronounced on the left, as well as right frontal degeneration. More recently, Philippi et al. (2012) reported positive correlations between hippocampal volume and episodic autobiographical memory for both recent and remote periods of life. Using the TEMPau task exploring autobiographical memory from three time periods (the last 5 years, middle age, teenage/childhood) and resting-state FDG-PET imaging, Eustache et al. (2004) reported a temporal gradient of recalls in favor of remote past and that right hippocampal metabolism correlated uniquely with recent memories. In addition, retrieval of recent memories was preferentially associated with right prefrontal cortex metabolism whereas remote memories relied more upon left prefrontal areas. According to the HERA model (Tulving et al., 1994), the right prefrontal cortex is more involved in episodic memory retrieval and the left in semantic memory retrieval. In this respect, the results reported by Eustache et al. (2004) support the idea that many autobiographical memories become “semanticized” over time and that preserved remote memories in AD patients are predominantly semantic by nature, even if some remote episodic memories can persist in these patients. In an fMRI study, Meulenbroek et al. (2010) contrasted the patterns of brain activity during retrieval of episodic autobiographical memories and during the processing of semantic information. They reported evidence of compensatory activations, notably within frontal areas, when AD patients retrieved episodic autobiographical memories. These compensatory activations in areas known to be involved in semantic processing, suggest that autobiographical memory would undergo an exaggerated shift from an episodic to a semantic content in AD.

Another aim of our study was to investigate the impact of sleep on the recall of autobiographical memories. Even if consolidation of freshly acquired memory traces can last months or years, it is well established that this process preferentially occurs during sleep and that the first post-learning night is crucial (Born et al., 2006). This has been demonstrated across a wide range of tasks for both procedural and episodic memories (Rauchs et al., 2005; Diekelmann and Born, 2010). In our laboratory, we have shown that both slow-wave sleep and Rapid-Eye Movement (REM) sleep are necessary for the consolidation of rich, vivid episodic memories. In particular, REM sleep favors the consolidation of the encoding context associated to the items (Rauchs et al., 2004). In addition, several studies indicate that sleep-dependent consolidation of episodic memories may be altered in older adults (Harand et al., 2012 for review) and we also reported correlations between sleep parameters and episodic memory performance in mild AD patients (Rauchs et al., 2008; Hot et al., 2011). Retention of recent personal episodic memories (e.g., memories of a recent conversation with a relative) was probed after sleep or an equivalent period of wakefulness in older adults (Aly and Moscovitch, 2010), but this issue has never been addressed in AD patients.

To sum up, episodic autobiographical memory is impaired in AD, even in the early stages of the disease (Murphy et al., 2008; Leyhe et al., 2009; Irish et al., 2010, 2011; Bastin et al., 2012). This deficit is mainly subserved by the dysfunction or degeneration of medial temporal areas. In most of the studies aforementioned, the exploration of the recent past was conducted on a period generally lasting about 1–5 years, completely neglecting the very recent past. In the present study, we explored episodic autobiographical memories experienced the day and the day preceding the assessment as well as memories dating back 2 years ago and the young adulthood period. The originality of this study also relies on the fact that our experimental design allowed us to control the events participants experienced and recalled, as they were present, most of the time, in the laboratory. A first aim of this study was therefore to precise, using resting-state ^18^FDG-PET, the neural substrates of retrieval of recent autobiographical memories. We also aimed at investigating the impact of sleep on the recall of autobiographical memories, and expected to find significant correlations between indices of sleep quality and/or quantity (such as time spent in slow-wave sleep or REM sleep) and episodic autobiographical memories.

## Materials and Methods

### Participants

Fourteen unmedicated, newly diagnosed AD patients (eight women, six men; mean age ± SD: 77.1 ± 4.1 years) with a MMSE score (Folstein et al., 1975) of 21 or higher (mean ± SD: 24.9 ± 2) participated in this study. Some data of most of these patients were previously published in two other studies (Rauchs et al., 2008; Hot et al., 2011). They were all recruited through a memory clinic, and all complained of memory impairment. They were selected on the basis of a neurological examination and a neuropsychological assessment, using the National Institute of Neurological and Communicative Disorders and Stroke and the AD and Related Disorders Association criteria for probable AD (McKhann et al., 1984). At the time of the study, none of the patients was being or had been treated with specific medication, such as antiacetylcholinesterase agents. None of them suffered from sleep disorders such as periodic limb movement disorder or sleep apnea, confirmed by polysomnography.

Autobiographical memory scores of AD patients were compared to those obtained in a group of 14 age-matched healthy controls (9 women, 5 men; mean age ± SD: 75.1 ± 4.6 years) recruited in clubs for retired people. They had no neurological or psychiatric disorders. The mean score (±SD) for the MMSE was 29.4 (±0.9). These subjects were also paired according to their level of education with AD patients.

All subjects were right-handed, native French speakers and gave their written consent to the study after detailed information was provided to them and to a member of their family. The study was done in-line with the Declaration of Helsinki following approval by the Regional Ethics Committee.

### General procedure

**Figure 1:**
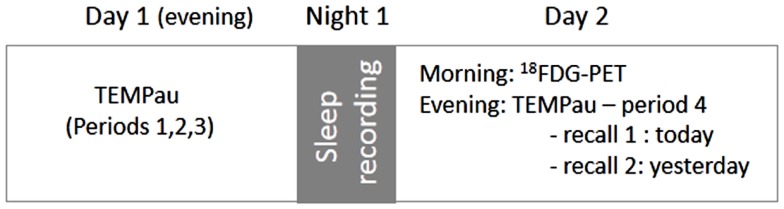
**Experimental design**. Period 1: young adulthood (18–30 years), period 2: last 2 years (except the last month), period 3: last month, period 4: today and yesterday. Note that only AD patients benefited the PET examination.

The general procedure is illustrated in Figure 1. Autobiographical memory for the very recent past (events experienced the day and the day preceding the assessment) was explored and compared to three other periods covering the young adulthood to the last month. On the first day, participants came to the hospital for a sleep recording. Before placement of electrodes, they performed the first part of the autobiographical memory (TEMPau) task assessing the three remote periods. Sleep was then recorded using standard polysomnography. The next morning, AD patients benefited an ^18^FDG-PET scan to measure brain glucose consumption at the resting-state. In the evening, the second part of the TEMPau task, assessing the very recent past, was proposed to patients and controls together with other episodic memory tasks not described here but whose results have been published elsewhere (Rauchs et al., 2007, 2008; Hot et al., 2011).

### Memory testing

We used an adaptation of the autobiographical memory task “TEMPau” developed by Piolino et al. (2003, 2009). This task, consisting of a semi-structured questionnaire, assesses the ability to recall detailed specific events situated in time and space from four time periods [P1: young adulthood (18–30 years), P2: the last 2 years (except the last month), P3: the last month, and P4: today and yesterday] as well as the subjective states of consciousness associated with those memories (Tulving, 1985, 2002).

For the most recent period (P4), participants had to recall what they did yesterday and today. More precisely, they were invited to relate a particular moment in the day, giving details about the chronology of the event, the place where it occurred and the people that were present.

For the three other periods, participants were invited to recall two personal events corresponding to two topics: (i) a meeting or an event associated to a person and (ii) a trip or journey. For each period, participants were invited to give details of a particular event. If a participant could not spontaneously recollect a specific event, cues were provided (for example, “on a day with a teacher or friend”), while he or she was encouraged to be specific if the memory was generic (e.g., “do you remember a particular day during this summer?”). After three cueing and/or encouragement attempts, the experimenter switched to the following topic or period.

Immediately after each recall, the participants were asked to indicate the subjective state of consciousness associated with the recall of *what* happened (i.e., the factual content), *where* (the place), and *when* (the moment). They were instructed to give a Remember, Know, or Guess response (Mäntylä, 1993) according to whether each of these three aspects of the recalled event was associated with conscious recollection, simply knowing, or guessing, respectively. A Remember response is defined as the ability to mentally relive specific aspects such as perceptions, thoughts, or feelings that occurred or were experienced at the time of the event. The participants were asked to give details aloud to ensure that they were using Remember responses properly. A Know response reflects simply knowing what happened, where and when, but this knowledge is not accompanied by any conscious recollection. A Guess response corresponds to aspects of the event that were neither consciously recollected nor simply known.

For the first three periods (P1, P2, and P3), the control of the veracity of the events recalled was made with the spouse/husband or a relative. For P4, we could perform a more stringent control of the events recalled by participants as they were present, most of the time, in the laboratory.

Each recalled event was scored on a four-point episodic scale based on that used by Baddeley and Wilson (1986). This scale takes into account the specificity of the memory (single or repeated event), the time, and spatial location, and the presence of details (perceptions, thoughts, or feelings). A specific event detailed and situated in time and space was given a score of 4 points. A specific event without any detail but located in time and space scored 3 points. A repeated or extended event scored 2 points or 1, depending on whether or not it was situated in time and space. Absence of memory or general information about the topic scored 0. The critical factor that allowed us differentiating specific events (scores 3 points) from a specific, detailed event (scored 4) was the failure, despite much encouragement, to add details concerning the source of acquisition. Recalled events scored 2 or 1 referred rather to personal semantic memory.

Two independent experts (GR and PP) rated each memory recalled and any difference of opinion between them was discussed until a consensus was reached. Two main scores were calculated for each period: (i) an overall autobiographical memory score, named hereafter “total recall score,” taking into account all types of recall, both specific and generic (maximum = 8 points for each period) and (ii) a strictly episodic recall referring to the recall of a specific memory, situated in time and space and with phenomenological details (perceptions, emotions, thoughts, mental images, …; maximum = 8 points for each period).

In addition, we also calculated a Remember score (*R*, maximum = 6 for each period) for the total number of *R* responses provided, irrespective of the kind of information (what, where, when) of each period and (iii) a justified Remember score (justified *R*, maximum = 6 for each period) for the number of *R* responses effectively associated with the recollection of a single event, with contextual details (thoughts, feelings, or perceptions for content, location for place, and time of day or temporal sequence for date).

### Sleep recording

For all subjects, sleep was recorded in the sleep laboratory using a Nicolet Acquisition System, including continuous recordings of EEG, electro-oculogram, electro-myogram recorded at the chin, and electrocardiogram. EEG activity was recorded from right and left central (C3/C4), temporal (T3/T4), and occipital (O1/O2) derivations of the extended 10–20 international system (Nuwer et al., 1999), using Ag/AgCl electrodes with a vertex ground and a right ear reference. The impedance for all electrode sites was kept below 10 kΩ. The EEG filter band pass was 0.03–35 Hz and was digitized at 125 Hz.

Three additional electrodes were placed at the outer canthus and supraorbitally to the right eye with a bipolar recording for electro-oculogram activity. Sleep recordings were scored by an experienced physician (FB) according to standard criteria (Iber et al., 2007). Total sleep time, sleep onset latency, sleep efficiency, and the time and percentage of time spent in each sleep stage were determined.

### PET methodology

In the morning of day 2, all the patients underwent a resting PET examination using [^18^F] Fluoro-2-deoxy-d-glucose (^18^F-FDG). Data were collected using the high-resolution PET device ECAT Exact HR+ with isotropic resolution of 4.6 mm × 4.2 mm × 4.2 mm (field of view = 158 mm). The patients were fasted for at least 4 h before scanning. The head was positioned on a headrest according to the cantho-meatal line and gently restrained with straps. ^18^F-FDG uptake was measured in the resting condition, with eyes closed, in a quiet and dark environment. A catheter was introduced in a vein of the arm for radiotracer administration. Following ^68^Ga transmission scans, 3–5 mCi of ^18^F-FDG were injected as a bolus at time 0, and a 10-min PET data acquisition period was begun at 50 min post-injection. Sixty-three planes were acquired with septa out (volume acquisition), using a voxel size of 2.2 mm × 2.2 mm × 2.43 mm (*x*, *y*, *z*). During PET data acquisition, head motion was monitored continuously with laser beams.

Preprocessing of FDG-PET data included (1) voxel-wise correction for partial volume effects (PVE) using the corresponding structural T1 MRI with the PMOD software (PMOD Technologies Ltd., Adliswil, Switzerland), (2) coregistration onto corresponding T1 MRI and spatial normalization to the MNI space using the parameters estimated from the corresponding T1-weighted MRI using the voxel-based morphometry 5.1 (VBM) toolbox (http://dbm.neuro.uni-jena.de) implemented in statistical parametric mapping 5 (SPM 5) software (Wellcome Trust Centre for Neuroimaging, London, UK), (3) quantitative scaling using the cerebellum gray matter as a reference to obtain standardized uptake value ratio (SUVr) images (cerebellum gray matter values were obtained for each participant using the cerebellum defined in the Automated Anatomical Labeling (AAL) atlas (Tzourio-Mazoyer et al., 2002), (4) smoothing with a 12-mm FWHM Gaussian kernel to blur individual variations in gyral anatomy and to increase signal-to-noise ratio, and (5) masking to exclude non-gray matter voxels. The resulting images were then used in the correlation analyses described below.

### Data analyses

Analyses focused on the recent past (P4), the three other periods being used as control data for behavioral analyses. Memory scores obtained with the TEMPau task were analyzed using analyses of variance (ANOVA) with group (AD vs. controls) as between-subject factor and periods (P1, P2, P3, and P4) as within-subject factor. These analyses were followed by *post hoc* tests (HSD Tukey) when applicable. As the two memories of the most recent period (P4) were experienced on two different days, separated by a night during which sleep was recorded, we conducted similar analyses dividing the period P4 in two sub-periods: yesterday and today. These analyses will allow to determine whether sleep has a beneficial effect on consolidation of autobiographical in AD patients and older adults. In addition, we also looked for correlations between sleep parameters (such as the percentage of time spent in each sleep stage) measured during the night and the memory score for the event experienced the previous-day.

Then, we investigated the neural substrates of recent memories in AD patients. To do so, correlations between the total recall score obtained for period P4 and brain metabolism were searched using SPM and Pearson’s correlation test. Only the positive correlations (i.e., in the neurobiologically expected direction) were assessed, using a statistical threshold (uncorrected for multiple tests) of *p* < 0.001 for the voxels, to limit the number of statistical tests and the attending risk of false positives.

## Results

### Autobiographical memory scores

Here is an example of what an AD patient related for the “yesterday” sub-period, describing his arrival at the hospital, the exploration of the remote period during the TEMPau task and the preparation for the sleep recording: “*Monday, in the afternoon, I prepared my belongings and the papers you need. I ate alone. I came to this hospital in the evening with my daughter. We came by car and left the house at about 7:45 pm. I performed some tests with you and we discussed about my life when I was a young man. It lasted more than 30 minutes. Then, two nurses (a man and a woman) put electrodes on my head. During the night, I found that the bed was too high and I felt cold*.”

The total recall score for this event was 3 (as it was a specific event, located in time and space, but without sufficient details). Indeed, he could not recall any details about the specific spatial context (the places where the different examinations occurred, his position in the room …), giving only general information (name of the city and the hospital). Thus, while the patient provided three Remember responses, only *R* responses associated with factual and temporal were justified.

**Figure 2:**
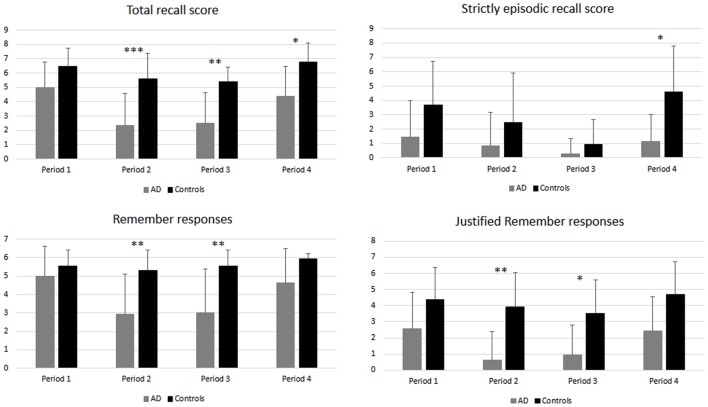
**Autobiographical memory scores in AD patients and healthy controls for the four time periods**. Stars indicate between group differences. *p < 0.05; **p < 0.01; ***p < 0.001.

Figure 2 illustrates the results for the total recall score, the strictly episodic recall score, as well as for the number of *R* and justified *R* responses in AD patients and controls across the four time periods. An ANOVA with period (P1, P2, P3, and P4) as within-subject factor and group as between-subject factor, performed on the total recall score revealed a significant main effect of group [*F*(1,25) = 31.5, *p* < 0.0001] and time period [*F*(3,75) = 10.8, *p* < 0.0001]. The group by period interaction was not significant [*F*(3,75) = 1.72, *p* > 0.17]. To further investigate the effect of time period, *post hoc* comparisons (HSD Tukey) were conducted and revealed that memory scores in both groups were not different for P1 and P4 (*p* > 0.97) and higher to those obtained for P2 and P3 (all *p* values < 0.002), indicating the existence of a reminiscence bump (P1) and a recency effect (P4). Performance on P2 and P3 did not differ significantly (*p* > 0.99). As we hypothesized a relative preservation of the very recent past in AD, we further investigated the effect of group according to the period. Group differences were observed for P2, P3, P4 (all *p* values < 0.05), patients scoring lower than controls, but not for P1 (*p* > 0.39).

A similar analysis conducted on the strictly episodic recall score revealed similar main effects [group: *F*(1,25) = 12.9, *p* < 0.001; period: *F*(3,75) = 4.99, *p* < 0.003] but no group by period interaction [*F*(3,75) = 1.7, *p* > 0.17]. *Post hoc* comparisons revealed that, in the whole group of participants, scores for P1, P2, and P4 were not significantly different (all *p* values > 0.26) while scores for P3, corresponding to the last month, were significantly lower than those for P4 (*p* < 0.006) and P1 (*p* < 0.02). A group difference was only observed for P4 (*p* < 0.05).

An ANOVA conducted on the number of Remember responses provided during memory recall revealed significant main effects of group [*F*(1,25) = 17.45, *p* < 0.001] and time period [*F*(3,75) = 5.75, *p* < 0.001] as well as a significant interaction between both factors [*F*(3,75) = 3.27, *p* = 0.026]. The number of Remember responses was stable across periods (all *p* values > 0.94) in controls, while AD patients provided more Remember responses for P1 and P4 (without any difference between them) than for P2 and P3 (all *p* values < 0.006, no difference between P2 and P3). Only scores for the two intermediate periods (P2, P3) significantly differed between groups (all *p* values < 0.01).

Finally, concerning the number of Remember responses justified by phenomenological details, the ANOVA revealed significant main effects of group [*F*(1,25) = 23.49, *p* < 0.001] and period [*F*(1,25) = 14.3, *p* = 0.005], but no interaction between these factors [*F*(3,75) = 0.83, *p* > 0.48]. A *post hoc* analysis conducted to further examine the effect of period revealed exactly the same pattern of results than for Remember responses (P1 = P4 > P2 = P3). Here again, only scores for the two intermediate periods (P2, P3) differed between AD patients and controls (all *p* values < 0.05).

**Figure 3:**
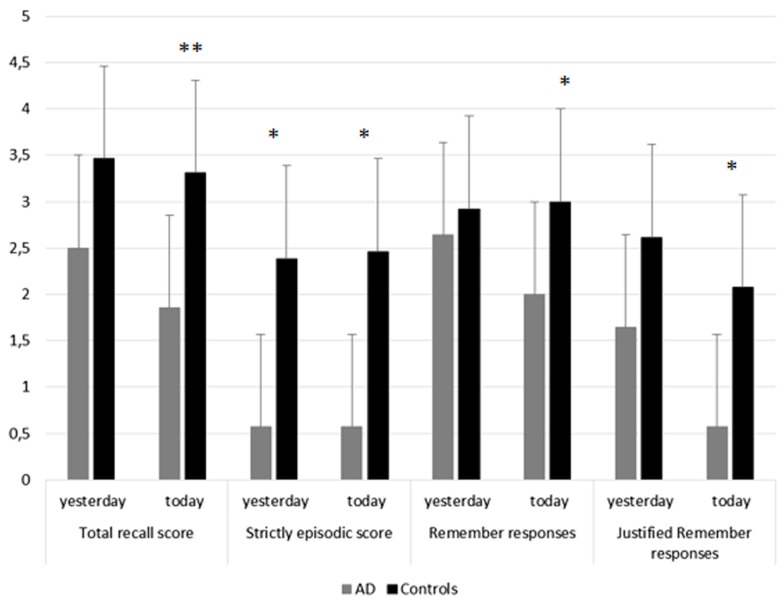
**Memory performance for yesterday and today events in AD patients and controls**. This figure illustrates performance in AD patients and controls for each memory score (total recall score, strictly episodic recall score, number of Remember responses, number of justified Remember responses) for the recent period (P4), distinguishing yesterday, and today sub-periods. Stars indicate between group differences. *p < 0.05; **p < 0.01.

Then, we conducted similar analyses dividing P4 into two sub-periods corresponding to yesterday and today events (Figure 3). These analyses revealed for the total recall score, the strictly episodic score and the number of Remember responses a main effect of group (all *p* values < 0.03), but no effect of sub-period or interaction between both factors. In contrast, for the number of justified Remember responses, we reported a main effect of group [*F*(1,25) = 9.3, *p* = 0.005] and a main effect of sub-period [*F*(1,25) = 8.7, *p* = 0.007]. The group by sub-period interaction was not significant [*F*(1,25) = 0.95, *p* > 0.33]. These results indicate that AD patients have lower scores than controls, and that in both groups, the number of justified Remember responses was higher for the events experienced the previous-day compared to today. These data suggest a beneficial effect of sleep on subsequent retrieval of recent autobiographical memories in mild AD patients.

### Sleep parameters

Sleep parameters in both groups are reported in Table 1. Group comparisons revealed that AD patients made significantly more sleep stage 1 than controls (*p* < 0.01) and tended to spent less time in slow-wave sleep (*p* = 0.072).

**Table 1:** **Sleep parameters in AD patients and controls**.

	AD patients (*n* = 13)	Controls (*n* = 14)
**GLOBAL PARAMETERS**		
Time in bed (min)	531.2 ± 77.1	495.3 ± 48.7
Sleep period time (SPT, min)	504 ± 79.1	477.9 ± 46.9
Total sleep time (TST, min)	387.3 ± 91.2	385.2 ± 68.5
Sleep latency (min)	27.3 ± 33.9	17.5 ± 19.8
Sleep efficiency (%)	72.3 ± 10.9	77.7 ± 10.5
WASO (min)	23.7 ± 10.1	19.6 ± 9.3
**SLEEP ARCHITECTURE (% SPT)**		
Stage 1	18.2 ± 5.4^a^	13.1 ± 4.4
Stage 2	26.7 ± 7.7	31.2 ± 7.5
SWS	18.7 ± 7.7^b^	23.5 ± 5.5
REM sleep	12.6 ± 6.5	12.6 ± 6

Sleep period time corresponds to time in bed – sleep latency.

SWS, slow-wave sleep.

^a^p < 0.01;

^b^p = 0.072.

### Correlations between autobiographical memory scores and sleep parameters

Finally, we searched for correlations, in AD patients, between sleep parameters and memory scores corresponding to events that occurred on day 1 (“yesterday” sub-period). Due to a technical recording problem, sleep scoring was not possible in one patient, which was removed from this correlation analysis.

**Figure 4:**
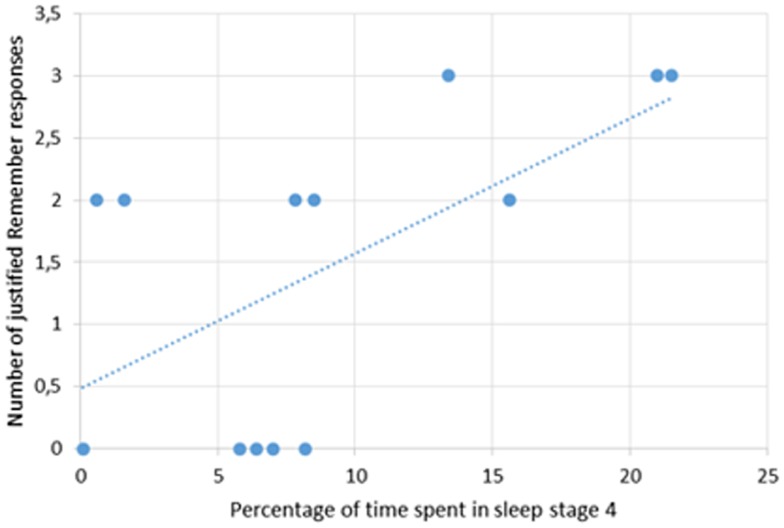
**Correlation between the number of justified Remember responses for the yesterday sub-period and the percentage of time spent in sleep stage 4 in AD patients**.

We observed significant positive correlations between the number of justified Remember responses and the percentage of time spent in slow-wave sleep (*r* = 0.55, *p* < 0.05), especially sleep stage 4 (*r* = 0.60; *p* < 0.05; Figure 4).

### Correlations between autobiographical memory and resting-state brain glucose consumption

**Figure 5:**
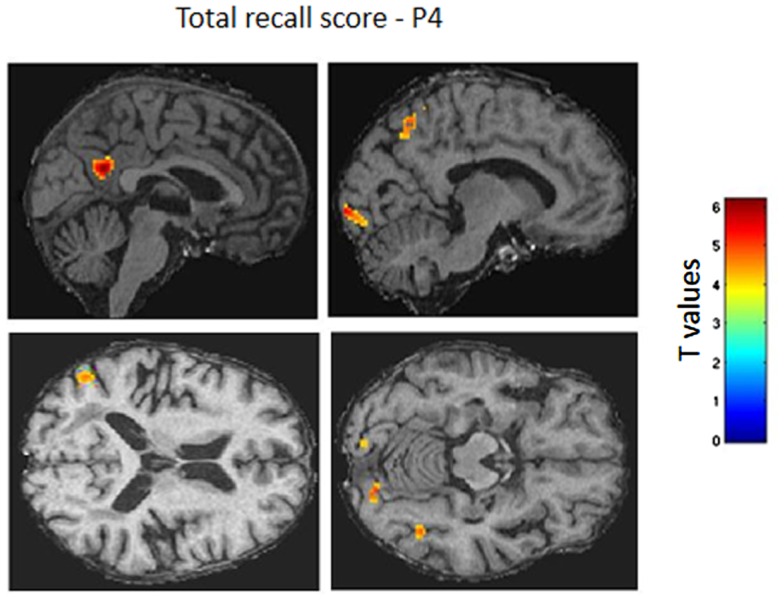
**Correlations between resting-state brain glucose consumption and autobiographical memory for recent events**. Positive correlations are shown as colored voxels superimposed on sagittal sections of an individual AD patient’s MRI normalized on the Montreal Neurological Institute (MNI) template (*p* < 0.001, uncorrected). The two sagittal sections (top) depict correlation with metabolism of the precuneus, calcarine region, and angular gyrus. The two axial sections (bottom) illustrate correlations with middle temporal gyri.

Correlations analyses between autobiographical memory scores and resting-state brain glucose consumption were conducted in the group of AD patients and only for the most recent period (P4). One patient did not benefit the PET examination and was excluded from these analyses. In addition, correlations were only searched for the total recall score due to a very limited inter-subject variability for the other measures. Thus, the total recall score positively correlated with brain glucose consumption in the precuneus bilaterally extending to the retrosplenial cortex, the calcarine region, the angular gyrus as well as middle temporal gyri (Table 2 and Figure 5). Then, we conducted similar analyses dividing P4 in two sub-periods corresponding to “yesterday” and “today.” The pattern of correlations between brain glucose consumption and memory scores for the “yesterday” period was very similar to that reported above. In contrast, for “today” events, correlations were only found in the precuneus but at a more permissive statistical threshold (*p* < 0.005).

**Table 2:** **Significant correlations between brain glucose consumption and the total recall score for the recent period (P4)**.

Neuroanatomical region	MNI coordinates (mm)			*k*	*Z*
	*x*	*y*	*z*
**P4 (INCLUDING THE TWO EVENTS)**					
Left precuneus	−2	−54	22	109	3.98
Right precuneus	2	−56	22	52	3.71
Left calcarine region	−10	−102	−6	74	3.66
Left angular gyrus	−52	−60	40	125	3.45
Left middle temporal gyrus	−52	−66	14	33	3.32
Right middle temporal gyrus	42	−56	14	30	3.41
**P4: “YESTERDAY” EVENTS**					
Right supramarginal gyrus	60	−14	28	56	3.67
Right lingual gyrus	18	−86	−14	40	3.61
Right precuneus	2	−56	20	34	3.61
Left inferior frontal gyrus	−56	20	6	18	3.54
Left calcarine region	−12	−104	−4	10	3.35
Right middle temporal gyrus	52	−66	6	27	3.29
Right inferior temporal gyrus	46	−38	−18	14	3.88
**P4: “TODAY” EVENTS**					
No significant correlation

*k* = *cluster size* > *10 voxels; correlations are reported at p* < *0.001 (uncorrected)*.

## Discussion

The present study was designed to investigate autobiographical memory in patients with mild AD, focusing on the very recent past. Compared to healthy controls, recall of autobiographical events was impaired in AD for all periods, excepted for the reminiscence bump period (P1). In addition, in AD patients, recall of very recent events (P4) was relatively better than for the periods covering the last 2 years and the last month (P2 and P3) and comparable to performance obtained for P1. Using resting-state PET imaging, we also revealed the brain areas subserving the retrieval of recent events in AD. Finally, we reported, for the first time, a correlation between the amount of slow-wave sleep and previous-day memory suggesting that the ability to consolidate episodic autobiographical memories is associated with sleep integrity.

The analysis of the total recall score revealed a temporally graded autobiographical amnesia in-line with previous reports (Kopelman, 1992; Nestor et al., 2002; Piolino et al., 2003; Hou et al., 2005). AD patients still exhibited a reminiscence bump, contrasting with a significant impairment for periods corresponding to the preclinical stages of their disease (the last 2 years). Furthermore, recall performance for the very recent past was comparable to that observed for the remote past, indicating that patients are still able to retain new personal specific information, provided the experienced events are relatively outstanding and different from typical daily events. Indeed, in the present study, patients had to recall an event that was new for them, coming to a different hospital than the one they used to visit for a sleep recording. However, when considering strictly episodic memories, patients were impaired for all periods, especially for the last month period (P3), confirming their altered capacity to recollect rich specific and unique personal episodes as reported in other studies conducted in Mild Cognitive Impairment (Tramoni et al., 2012) and in AD patients (Eustache et al., 2004; Martinelli et al., 2013b). The comparison of older adults and AD patients suggest that the content of autobiographical memories integrates more semantic, general information, and only few episodic details, as previously stated by Meulenbroek et al. (2010).

Recollective experience was also disrupted in AD patients as attested by the significant decrease in the number of Remember responses – justified or not-, fitting nicely with other studies (Piolino et al., 2003; Rauchs et al., 2007; Irish et al., 2010, 2011; Tramoni et al., 2012). However, for recent and remote memories (P4 and P1), patients still have a feeling a mentally reliving the events as attested by the lack of significant group differences for Remember and justified Remember responses. Some old memories can remain very vivid, even in AD patients, because they are particularly important for the subject’s identity or are emotionally laden. For the very recent past, in this study, patients experienced events that were different from their everyday life, and therefore may be more resistant to forgetting. Our data indicate that they can have the feeling to relive these events, but cannot retrieve as much episodic details as healthy aged subjects. However, interestingly, patients provided significantly more Remember responses justified by phenomenological details for the recall of the events that occurred yesterday compared to those that occurred earlier during the day. This suggests that sleep, even if it is also disturbed in AD patients (Petit et al., 2004; Beaulieu-Bonneau and Hudon, 2009), can strengthen memory traces and reduce their sensitivity to forgetting. This point will be specifically discussed later.

Then, we looked at the brain areas subserving the recall of recent autobiographical memories in AD patients. To do so, we searched for correlations between autobiographical memory scores obtained for the period P4 and resting-state measures of brain glucose consumption. These analyses revealed that total recall score for very recent events was related to the metabolism of posterior cortical areas, including the precuneus and retrosplenial cortex and the calcarine region, the angular gyrus and lateral temporal areas, mainly on the left side. These regions play a central role in the retrieval of episodic autobiographical memories (Svoboda et al., 2006; Cabeza and St Jacques, 2007; Martinelli et al., 2013a), even in older adults (Viard et al., 2007, 2010). The retrosplenial cortex is early and severely hypometabolic in the course of AD (Nestor et al., 2003) and its activity has been related to episodic memory loss in AD (Desgranges et al., 2002). Furthermore, several studies showed greater activation of this area for recent compared to remote autobiographical memories in healthy subjects (for review, Cabeza and St Jacques, 2007). Several accounts have been suggested to explain the more important role of this region, together with the calcarine area, in recent autobiographical memories, including the construction of generic visual representations, retrieval of personally familiar information, emotional processing, and vivid recollection (Cabeza and St Jacques, 2007).

Correlations were also found in lateral temporal areas. In healthy subjects, the left middle temporal gyrus may subtend the access to general information in-line with the constructivist model of autobiographical memory proposed by Conway (Conway and Pleydell-Pearce, 2000). Indeed, according to this model, memories are not stored as a perfect record of the original event, but are rather reconstructed from our autobiographical knowledge stores. Recollecting a specific autobiographical memory therefore requires to access first general information before retrieving more precise and specific elements. Viard et al. (2007) reported, in healthy aged subjects, an activation of lateral temporal areas only for the young adult period, suggesting that the reconstruction process occurs mainly for remote memories. In AD patients, however, the correlation observed between autobiographical memory scores for the recent past and left temporal activity suggest that they use this reconstructive process even when retrieving very recent events.

This pattern of correlation was observed for events that occurred yesterday and less for those that occurred the day of the examination. It suggests that these areas play a role in the retrieval of autobiographical memories that were already remodeled and reorganized within neural networks, in particular during sleep episodes, even if the consolidation process is not necessarily completed.

Whole brain regression analyses (but also region-of-interest analyses, data not shown) failed to reveal any significant correlation between memory scores and hippocampal activity, even at a more lenient statistical threshold. Given the fact that the hippocampus is one of the primary sites of AD pathology (Baron et al., 2001) and in light of a previous study conducted in our laboratory and revealing a correlation between hippocampal metabolism and autobiographical memory scores for the recent past (Eustache et al., 2004), this finding may appear unexpected. However, the recent period in Eustache et al. (2004) study covered the last 5 years and is therefore very different from the recent past explored here (yesterday and today), making the comparison between the two studies tricky. Another study using also the TEMPau task failed to reveal significant correlations between hippocampal metabolism and autobiographical memories experienced during the last 12 months, suggesting that the decline in autobiographical memory in early stages of AD may be due to a dysfunction of other brain regions within the autobiographical core brain network (Bastin et al., 2012), such as the posterior cingulate cortex, already associated to the decline of episodic memory performance (Chételat et al., 2003; Bastin et al., 2010), and the precuneus, involved in visual imagery (Fletcher et al., 1995). In our study, correlations were observed in some of these areas, notably the precuneus but also in the temporo-parieto-occipital junction. We mentioned above that the events experienced by patients during the period P4 were different from their everyday life, probably more emotional, and therefore may be more resistant to forgetting. Amygdala atrophy is prominent in early AD (Baron et al., 2001; Basso et al., 2006; Poulin et al., 2011). However, a beneficial effect of emotion on memory was reported in AD patients in some studies (e.g., Boller et al., 2002; Nieuwenhuis-Mark et al., 2009; Borg et al., 2011, see also Klein-Koerkamp et al., 2012 for review), albeit not consistently (Abrisqueta-Gomez et al., 2002; Kensinger et al., 2002, 2004). We surmise that the emotional valence of these recently experienced events together with the fact that they were particularly self-relevant and uncommon may have favored their encoding and consolidation.

The present study also aimed at investigating, for the first time, the impact of sleep on the quality of recall of recent autobiographical events. First, the two events of the very recent past period (P4), corresponding to what the patients did today and yesterday, were compared. This analysis disclosed better memory performance in AD patients, at least for the number of justified Remember responses, for events experienced the previous-day compared to those experienced the day of the assessment. The time elapsed between the encoding of memories and retrieval was much shorter for the same day memory (<12 h) than for the previous-day memory (about 24 h, with a night of sleep in-between). Thus, the difference observed between the two sub-periods could be due either to the fact that the retention interval was longer (but which is generally supposed to induce greater forgetting) or to a genuine effect of sleep. Further studies comparing memory retrieval after equal retention intervals filled with sleep (naps for instance) and wakefulness are further needed to disentangle this issue. However, we observed a positive correlation between the number of justified Remember responses and the percentage of time spent in slow-wave sleep, especially sleep stage 4. These results indicate that patients still exhibiting high amounts of slow-wave sleep have relatively better memory recollection that those whose sleep is disrupted. It also confirms that sleep disturbances in AD are not a secondary symptom of the disease, but can really worsen the cognitive performance, in-line with previous studies (Rauchs et al., 2008; Hot et al., 2011; Westerberg et al., 2012). The positive correlation with slow-wave sleep indicates that, despite an impairment of autobiographical memory especially for the period covering the last 2 years, consolidation of episodic autobiographical mnesic traces overnight is not totally disrupted at this stage of AD. These data are likewise reminiscent and extend those reported by Aly and Moscovitch (2010) in healthy older adults, showing that brain mechanisms supporting sleep-dependent memory consolidation may be preserved when the studied material engages one’s interest.

In a previous study investigating, in young adults, the effect of sleep and sleep deprivation on consolidation of episodic memories assessed by means of a word-list learning task, we showed that both slow-wave sleep and REM sleep are involved in the consolidation of rich, vivid episodic memories and that REM sleep favored preferentially the consolidation of spatial information and of details about the encoding context (Rauchs et al., 2004). This result was not replicated in the present study. Interestingly, sleep macrostructure in our group of AD patients only slightly differed compared to healthy aged controls, but both groups exhibited a marked decrease in the amount of slow-wave sleep compared to young adults. The positive correlation between justified Remember responses and time spent in slow-wave sleep suggest that the decrease in this sleep stage, classically observed in older adults, and even more in AD patients, would compromise the initial steps of memory consolidation and would prevent processes occurring normally during subsequent REM sleep to consolidate contextual details. Interestingly, Westerberg et al. (2012) also reported correlations between memory performance assessed using a word-pair recall task, and indices relative to slow-wave sleep (delta power in Non-REM sleep) in MCI patients.

To conclude, the present study shows that autobiographical memory is altered in AD patients across the entire lifespan, but with a relative preservation of memories that occurred during the very recent past. Retrieval of very recent events relies upon the functional integrity of the precuneus and retrosplenial cortex, the middle temporal cortex, and visual areas, reflecting at the same time access to general information, reconstruction, and visual imagery processes as well as recollection of specific details. Finally, we provide the first evidence of an association between the ability to recall recent autobiographical memories and preceding slow-wave sleep, highlighting the utmost importance of preserving sleep quality in older adults for optimal cognitive functioning.

## Conflict of Interest Statement

The authors declare that the research was conducted in the absence of any commercial or financial relationships that could be construed as a potential conflict of interest.
